# Prevalence and Barriers to Lung Cancer Screening in Karachi, Pakistan: A Cross-Sectional Survey of Smokers and Physicians

**DOI:** 10.7759/cureus.1248

**Published:** 2017-05-15

**Authors:** Aleeza Abbasi, Rabbia Siddiqi, Aatika Owais, Tooba Laeeq, Sara N Ali, Zonaira Mushahid, Syed M Ahsan, Aliya S Jatoi, Aleena Abbasi, Ifrah Butt, Ruba Ali, Maham Abbasi, Syeda Naintara N Jaffri, Mariam Jabir, Hajra Khanani, Kaneez Fatima

**Affiliations:** 1 Dow Medical College, Dow University of Health Sciences (DUHS), Karachi, Pakistan; 2 Department of Internal Medicine, Dow University of Health Sciences (DUHS), Karachi, Pakistan

**Keywords:** smoking, lung cancer, barriers to healthcare, barriers to screening, lung cancer screening

## Abstract

**Background:**

Early detection of lung cancer using low-dose computed tomography (LDCT) can potentially reduce morbidity and mortality. However, LDCT for lung cancer screening, especially in low income countries, has been underutilized. The objective of this study was to evaluate the prevalence and the potential personal, social, and economic barriers of lung cancer screening using LDCT.

**Methods:**

A total sample of 156 smokers and 200 general physicians was collected during December 2016-February 2017 from community settings in Karachi, Pakistan. Two separate questionnaires were constructed to characterize participants’ knowledge, attitudes, and practices regarding lung cancer screening. Screening-eligible smokers and physicians were asked to identify patient barriers to screening and were asked their opinion regarding most effective approach for increasing awareness of screening guidelines.

**Results:**

The majority of smokers' (n=91, 58.3%) and physicians' (n=131, 65.7%) beliefs about the US Preventive Services Task Force (USPSTF) eligibility criteria were inconsistent with the actual recommendations. Major barriers to screening included financial cost, lack of patient counseling and health anxiety related to screening. Over two-thirds (n=105, 67.3%) of smokers were receptive to further information about LDCT screening, and half (n=78, 50.0%) favored one-on-one counseling by their physician, compared to other media. Only one-third (n=65, 33.3%) of physicians reported use of LDCT screening, although 54.5% (n=108) felt that screening implementation would be very effective in their practice.

**Conclusion:**

LDCT screening is currently an uncommon practice in Pakistan. Financial cost, inadequate doctor-patient communication, and lack of awareness of guidelines among both patients and physicians are the major barriers in the utilization of LDCT screening.

## Introduction

Lung cancer remains the leading cause of cancer mortality worldwide. In 2015, approximately one-fifth of cancer-related deaths were due to lung cancer, and a quarter of all cancer deaths were attributed to tobacco use [1]. The overall five-year survival rate for invasive lung cancer is only 17.7%, however for localized disease, this proportion is much higher (55%) [2]. Since disease stage at the time of clinical diagnosis is the most important determinant of prognosis, efforts are being made to increase early detection of lung cancer. Results of the 2011 National Lung Screening Trial showed a 21% reduction in lung cancer mortality following annual screening of high-risk individuals with low-dose computed tomography (LDCT) [3]. Currently, the United States Preventive Services Task Force (USPSTF) recommends annual LDCT screening for individuals aged between 55 and 80 years having a minimum smoking history of 30 pack-years, who either currently smoke or have quit within the past 15 years, and who do not have a prior malignancy [4].

Following a growing interest in determining the feasibility of implementing LDCT screening according to USPSTF guidelines, a number of studies have investigated patient and physician barriers to screening. These studies identified several barriers to LDCT screening including inadequate knowledge of screening recommendations among physicians, and financial cost and concerns of radiation exposure, on the part of smokers [5-9].

The above mentioned studies are representative of affluent countries of the Western world. However, a search on the PubMed database with keywords "lung cancer screening barriers" did not yield any results for relevant studies conducted in Pakistan, or even in South Asia. As has been observed with other cancers, notably breast and cervical, barriers to screening in developing countries are distinct from those in developed nations [10]. In low income and low education settings, for instance, financial cost and cultural beliefs are more important deterrents to screening [11]. Similarly, in these populations, fear of unnecessary radiation exposure is unlikely to be a relevant issue. It is therefore warranted for research to focus additionally on barriers specific to low income countries, such as Pakistan.

The primary objective of our study was to characterize the prevalence and potential barriers to LDCT lung cancer screening in Karachi, Pakistan. We also aimed to determine the perceived efficacy and safety of LDCT screening among primary-care physicians and screening-eligible smokers. A secondary objective was to identify ways to improve effective lung cancer screening practices in high-risk smokers.

## Materials and methods

### Study population

In this cross-sectional study we surveyed high-risk smokers and primary-care physicians in Karachi, Pakistan to assess their perception of LDCT screening for lung cancer. The study was approved by the institutional review board of Dow University of Health Sciences.

Current or former smokers who met the USPSTF eligibility criteria for annual low-dose CT lung cancer screening were recruited in our study. We employed purposive sampling using community-based recruitment methods. Individuals who were seen to be smoking in a variety of public places such as parks, shopping malls, and hospitals were approached. We also inducted eligible smokers based on referrals from existing participants in the study. In order to avoid interviewer bias, questionnaires were self-completed wherever possible. For participants who were unable to read or were not comfortable with English, interviewers were employed. The interviewers selected were fluent in Urdu (the local language) and were given prior training on how to ask questions in a neutral and professional manner. Those smokers who did not provide consent, and those with existing lung or other malignancy were excluded.

Primary-care physicians from both private and public healthcare settings were approached in person and were administered a paper-based standardized questionnaire if they gave consent. Physicians who had less than one year of clinical experience or those who did not spend time in direct patient care were excluded. Of 228 general physicians approached, 28 did not respond, giving a response rate of 71.4%.

### Data collection and analysis

Data collection continued from December 2016 through February 2017. Data was entered in Statistical Package for the Social Sciences (SPSS) v 22 (IBM, NY, USA), and descriptive statistics were calculated for categorical responses. We excluded missing values from our analysis.

### Questionnaire details

Survey questions were developed from existing literature regarding LDCT lung cancer screening practices [12-13] and adapted to the local social and health context. Both questionnaires were reviewed for relevance and clarity by two doctors.

The questionnaire to smokers consisted of four sections. The first section noted demographic variables and self-reported smoking status and smoking history. The second section assessed the respondents' knowledge of lung cancer screening guidelines generally as well as specifically of LDCT screening and their perceived benefit of early detection of lung cancer. In the third section a list of statements such as “You’re afraid of a positive result” was provided, and respondents were asked to identify each as a “barrier” or “not a barrier” to their participation in screening practices. The final section assessed their receptiveness to lung cancer screening awareness and preferred mode of learning (e.g. via newspapers, online infomercials, or face-to-face discussions).

The questionnaire to physicians recorded demographic details such as age, sex, years of clinical practice, and percentage of patients who smoke cigarettes. Five close-ended questions gauged the physicians' knowledge of the USPSTF eligibility criteria for lung cancer screening in smokers. Physicians were asked to rate six factors as a "major barrier," "minor barrier," or "not a barrier" to patient participation in lung cancer screening. The final three questions enquired about their screening and counseling practices in patients with a significant smoking history and recorded their recommendations for screening implementation and its feasibility in Pakistan.

## Results

### Smokers eligible for screening

Demographics

The mean age of our sample of 156 screening-eligible smokers was 57.3 years, and the mean age at which they started smoking was 21.9 years. Other characteristics are provided in Table 1.

**Table 1:** Summary of demographic characteristics and self-reported smoking status of smokers

	Frequency	Percentage (%)
Gender		
Male	155	99.4
Female	1	0.6
Education status		
Illiterate	39	25.7
Primary (Grade 1-5)	12	7.9
Secondary (Grade 6-12)	47	30.9
Tertiary (Graduate level and beyond)	54	35.5
Marital status		
Single	9	6.0
Married	140	94.0
Residence		
Urban	114	76.0
Rural	36	24.0
Smoking status		
Current smoker	121	77.6
Former smoker	35	22.4
Chewing betel nuts		
Never	108	69.2
Past	22	14.1
Current	26	16.7
Chronic disease status		
Hypertension	25	16.0
Diabetes	14	9.0
Heart disease	6	3.8
Other chronic illness	3	1.9
≥1 of the above	37	23.7

Knowledge of Screening Recommendations

Only one-third (n=52, 33.3%) of smokers had heard of lung cancer screening, although 58.3% (n=91) did agree that early detection of lung cancer could possibly benefit them. The majority (n=107, 68.6%) did not know whether low-dose CT was effective in screening for lung cancer and were also not sure if LDCT was a safe modality (n=108, 69.2%). When briefed about lung cancer screening, 61.2% (n=93) felt that all smokers should be screened regardless of smoking status (current or former smoker) and smoking history.

Patient Barriers to Screening

Barriers to screening according to smokers are summarized in Table 2. Generally, most smokers agreed that health anxiety related to screening (n=107, 68.6%) and financial cost (n=94, 61.4%) were among their reasons for not getting screened. Most did not think that the tests were unsafe (n=110, 73.3%) and did not report being afraid of hospitals (n=95, 60.9%). Of the respondents, 52.6% (n=82) stated that a fear of positive results prevented them from getting screened. For 47.4% of smokers (n=73), the belief that treatment is more of a suffering than the disease itself, contributed to the lack of participation in screening efforts.

**Table 2:** Patient barriers to screening as identified by smokers eligible for screening

		Frequency (n)	Percent (%)
Lung cancer screening is too expensive for you to afford			
	Yes	94	61.4
	No	59	38.6
You’re afraid of a positive result			
	Yes	82	52.6
	No	74	47.4
If screening results are negative, it is alright to continue smoking			
	Yes	55	35.5
	No	100	64.5
You think you are too old to benefit from screening			
	Yes	56	35.9
	No	100	64.1
You think the tests are unsafe			
	Yes	40	26.7
	No	110	73.3
Screening would only make you feel more anxious about your health			
	Yes	107	68.6
	No	49	31.4
Fear of hospitals and CT scanners prevent you from screening			
	Yes	61	39.1
	No	95	60.9
The treatment is more of a suffering than the disease itself			
	Yes	73	47.4
	No	81	52.6

Attitudes and Practices Related to Screening

**Figure 1:**
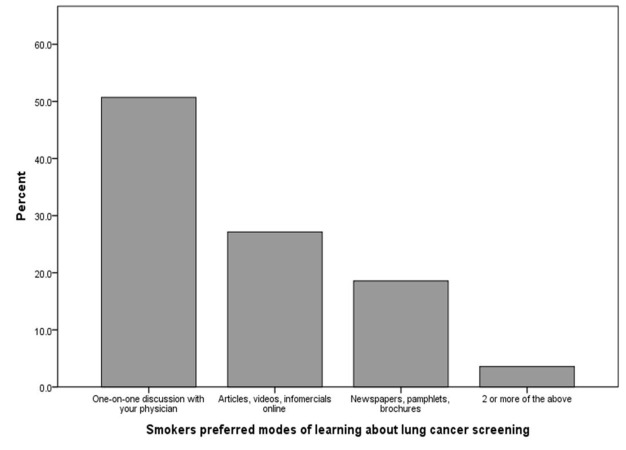
Preferred modes of learning about lung cancer screening as indicated by smokers

Most smokers had not previously received LDCT screening for lung cancer (n=132, 84.6%) or any other screening test (n=119, 76.3%). However, two-thirds (n=105, 67.3%) indicated that they would like to know more about lung cancer screening. By far the most popular (n=78, 50.0%) means of spreading awareness to smokers regarding LDCT screening was one-on-one discussions with primary-care physicians (Figure 1).

### Primary care physicians

Demographics

Our final sample included 200 primary-care physicians, of whom 43.5% (n=87) were female. The mean age of respondents was 37.7 years while the mean experience was 10.9 years in primary-care practice. About half of the respondent physicians (n=99, 50.3%) estimated the frequency of smokers in their patient population to be ≥50%, while another 35.5% of physicians (n=70) estimated this frequency to be 25-49%. A minority (n=28, 14.2%) indicated that less than 25% of their patients were smokers.

Knowledge of Screening Recommendations

In this study, 55.3% of physicians (n=110) correctly identified LDCT as the most effective lung cancer screening modality, with the rest favoring: chest X-ray alone (23.6%); chest X-ray plus sputum cytology (18.1%); sputum cytology alone (2.0%); and “don’t know” (1.0%). One-fifth (n=39, 19.8%) of the physicians knew that a minimum of 30 pack-year history was required for screening eligibility, and many believed that the minimum was 20 pack-years (n=75, 38.1%) or 10 pack-years (n=47, 23.9%). Over one-fourth (n=53, 26.5%) of the physicians in our study were aware of the minimum smoker age for screening eligibility, but only 9.1% (n=18) of them correctly indicated 80 years as the maximum age beyond which annual LDCT screening is not recommended. We found that 52.5% (n=104) believed that there was no upper age limit to lung cancer screening. The majority (n=137, 69.2%) agreed that the frequency of screening in high-risk patients should be once a year.

Perceived Barriers to Screening

The financial cost of annual LDCT screening and a lack of patient counseling were most frequently (n=140, 70.4% and n=127, 64.1%, respectively) identified by physicians as "major barriers" to patient participation in lung cancer screening (Table 3). Fear of positive results was considered to be a "major barrier" by 40.6% (n=80) physicians, and a "minor barrier" by another 47.2% (n=93) physicians. A lack of perceived benefit of screening was largely thought to be a "minor barrier" (n=102, 51.5%). The perceived health risks of LDCT screening and the unacceptability of screening methods were mostly either not recognized as barriers to screening (n=54, 27.1% and n=51, 25.8%, respectively) or were seen as "minor barriers" (n=90, 40.5% and n=91, 46.0%, respectively).

**Table 3:** Perceived barriers to lung cancer screening as rated by primary-care physicians

		Frequency (n)	Percent (%)
Financial cost	Major barrier	140	70.4
	Minor barrier	42	21.1
	Not a barrier	16	8.0
	Don’t know	1	0.5
Lack of patient counseling	Major barrier	127	64.1
	Minor barrier	60	30.3
	Not a barrier	9	4.5
	Don’t know	2	1.0
Lack of perceived benefit of screening	Major barrier	64	32.3
	Minor barrier	102	51.5
	Not a barrier	23	11.6
	Don’t know	9	4.5
Fear of positive results	Major barrier	80	40.6
	Minor barrier	93	47.2
	Not a barrier	20	10.2
	Don’t know	4	2.0
Perceived risk of screening (e.g. concerns of radiation exposure)	Major barrier	47	23.6
	Minor barrier	90	45.2
	Not a barrier	54	27.1
	Don’t know	8	4.0
Unacceptability of screening methods (e.g. fear of CT scanner)	Major barrier	54	27.3
	Minor barrier	91	46.0
	Not a barrier	51	25.8
	Don’t know	2	1.0

Attitudes and Practices Related to Screening

**Figure 2:**
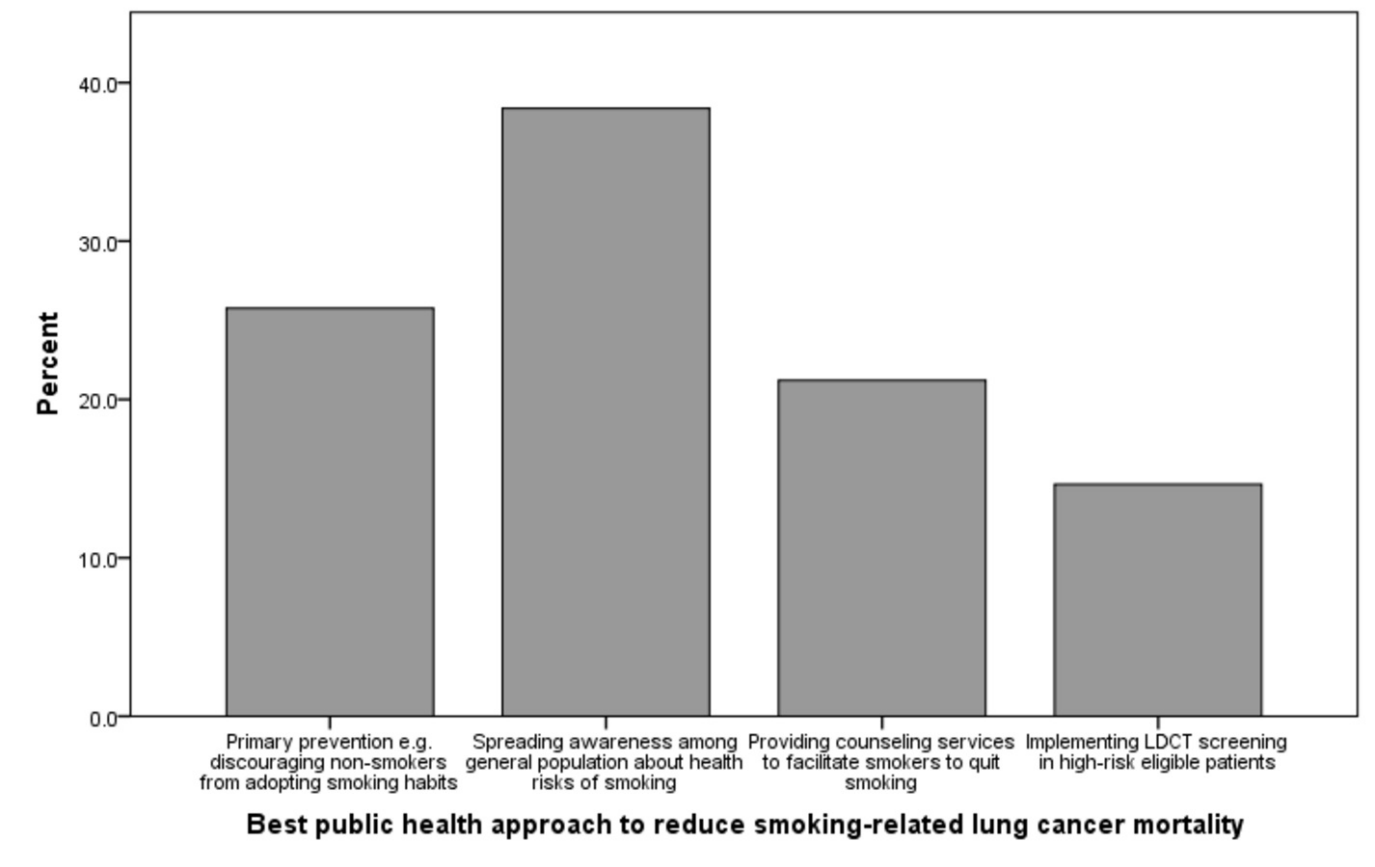
Best long-term public health approach to reduce smoking-related lung cancer mortality in Pakistan according to primary-care physicians

One-third physicians (n=65, 33.3%) reported that over the past 12 months they had at least once ordered LDCT screening for asymptomatic patients with a significant smoking history. Also, 63.1% (n=125) additionally indicated that they had discussed the risks and benefits of lung cancer screening with high-risk patients. The majority (n=172, 86.9%) had also counseled patients about smoking cessation strategies in the past one year. As shown in Figure 2, increasing health awareness in the general population regarding smoking emerged as the most popular public health approach to reduce long-term smoking-related lung cancer mortality. Furthermore, 54.5% (n=108) physicians felt that screening implementation would be very effective in their patient population, 44.4% (n=88) felt that it would be somewhat effective, and only one percent (n=2) felt that it would have no effect at all.

## Discussion

### Prevalence of LDCT screening

We found a low prevalence of lung cancer screening in Karachi, Pakistan. Only one-third of the smokers in our study had heard about lung cancer screening, implying a lack of awareness among the general population. Most smokers in our study had not previously received LDCT or any other screening tests for lung cancer. Furthermore, although more than half the physicians correctly identified LDCT as the best modality for lung cancer screening, only one-third of the participating physicians had at least once recommended LDCT in the past 12 months, despite a significant number of their patients being heavy smokers. This implies that certain barriers to effective lung cancer screening exist in Karachi. A study by Jemal, et al. reported similar findings in the US [14].

### Barriers to LDCT screening

Our study identified financial cost as the major barrier to screening in high-risk smokers in Pakistan. Previous literature shows that lung cancer screening with LDCT at the patient’s own expense can result in decreased intention to undergo screening and a lower attendance at follow-up visits [15]. The estimated cost of an initial LDCT scan is Rs 10,000-1,000,000 in Pakistan (USD 99-1000) [16]. This would be a huge financial burden for the majority of our population, who fall below the poverty line and for whom Rs. 10,000 is more than a month’s salary. Health insurance services are out of reach for most, which may contribute to the concern of cost as an important barrier to screening.

Health anxiety with respect to screening also emerged as a major barrier in our study. Fear of positive results was cited as another barrier to patient participation in lung cancer screening. However, contrasting results have been found in previous studies regarding the fear factor, with some studies linking fear to poorer screening while others link it to more frequent screening for cancer [17]. Another factor found to be a major barrier in our study was a lack of counseling regarding LDCT. In a recent study, time constraints were cited by primary care physicians as a reason for the lack of appropriate counseling and shared decision making for lung cancer screening [8]. This especially holds true for public sector settings in Pakistan, where already-overworked doctors are required to cater to large numbers of patients in a limited time frame, making each doctor-patient interaction necessarily brief. As a result, these physicians are unable to engage in extensive patient counseling efforts.

Inadequate knowledge of the physicians was also divulged in our study, as an overwhelming majority of the physicians were not aware of the upper and lower age limit and the minimum pack-year history of patients who should be recommended LDCT. USPSTF has developed a concise information sheet on lung cancer screening for health care providers which targets to bridge the knowledge gap [18]. Fear of hospitals and CT scanners and concerns about the safety of LDCT were not found to be significant barriers to patient participation in screening. This is in contrast to a previous study [19] where it was found that fatalistic beliefs, fear of radiation exposure, and anxiety related to CT scans were significantly associated with decreased intention to screen. This may be because the underprivileged and uneducated majority here is generally oblivious to how a CT scanner works or even what it looks like, in some cases leading study participants to deny the sentiment altogether. Also, many people hold the view that since these tools are designed to help the doctor in treating them, it is needless to fear them.

### Recommendations and future directions

Our study found that the most popular means of spreading awareness among smokers about screening was one-on-one discussion with the physician. To this end, fostering better doctor-patient interaction and encouraging doctors to carry out extensive counseling is essential. Nhung, et al. carried out a study among Korean men in 2015, which validated that increased discussion with patients regarding the benefits of LDCT screening improved screening participation from 10% to 95% [20]. Most physicians in our study felt that the best public health approach to prevent smoking-related lung cancer mortality was to spread awareness regarding risks of smoking, again highlighting the gap in communication between primary care physicians and smokers who are eligible for screening.

A majority of physicians recommended the frequency of screening by LDCT to be once annually. This is in accordance with the USPSTF recommendation. However, as discovered in another study [21], biennial screening is just as effective as annual screening. Moreover, biennial screening may save about one-third of LDCT scans [21]. Hence, biennial screening can be taken as a minimum frequency, making screening less expensive and more achievable and therefore, can be helpful in increasing patient participation.

We recommend that in our socio-economic setting, measures be put in place that spread awareness among the at-risk population regarding LDCT as the recommended screening modality for lung cancer. Further research should investigate the barriers to appropriate patient counseling by physicians. According to one study, taking the cognitive aspects of participation into account increases the uptake of lung cancer screening practices among high-risk smokers [22]. Increased government funding for LDCT screening for eligible smokers can allow those smokers to get screened, who cannot otherwise afford it. However, this remains controversial as some postulate that increasing insurance coverage for screening may encourage smokers to continue smoking without fears of its negative heath consequences. As stated in the previously mentioned study [22], ‘effective smoking cessation interventions delivered in the LDCT setting could be a highly cost‐effective way to reduce smoking‐related morbidity and mortality’. A cost-utility analysis indicated that offering smoking cessation interventions along with annual screening would increase the cost effectiveness of lung cancer screening by 20-45% [23].

### Limitations

Our study has several limitations that need to be considered. Convenience sampling was employed along with self-reporting in collecting data. Some of the respondents could not recall their ages and the exact year when they started smoking, which may have led to recall bias. There were no open-ended questions in the questionnaires, thus limiting the extent to which physicians and smokers could communicate their opinions. Lastly, all but one smoker in our study were male. This does not represent the number of male and female smokers in the general population. Future studies should address these limitations.

## Conclusions

In conclusion, LDCT screening is currently an uncommon practice in Pakistan, despite the burden of smoking-related lung cancer deaths. Financial cost, inadequate doctor-patient communication and lack of awareness of guidelines among both patients and physicians are the major barriers in the utility of LDCT screening. Approaches to overcome these barriers and implement effective screening practices include improving patient counseling and increasing the affordability of LDCT screening services.
